# Pregnancy related anxiety and general anxious or depressed mood and the choice for birth setting: a secondary data-analysis of the DELIVER study

**DOI:** 10.1186/s12884-016-1158-7

**Published:** 2016-11-22

**Authors:** A. B. Witteveen, P. De Cock, A. C. Huizink, A. De Jonge, T. Klomp, M. Westerneng, C. C. Geerts

**Affiliations:** 1 Department of Midwifery Science, AVAG and the EMGO Institute for Health and Care Research, VU University Medical Center, Van der Boechorststraat 7, 1081 BT Amsterdam, The Netherlands; 2 Department of Developmental Psychology, VU University Amsterdam, Amsterdam, The Netherlands; 3 EMGO+ Institute for Health and Care Research, VU Medical Center, Amsterdam, The Netherlands; 4 Department of Clinical Child and Family Studies, VU University Amsterdam, Amsterdam, The Netherlands

**Keywords:** Pregnancy, Anxiety, Mood, Planned place of birth, Midwife-led care

## Abstract

**Background:**

In several developed countries women with a low risk of complications during pregnancy and childbirth can make choices regarding place of birth. In the Netherlands, these women receive midwife-led care and can choose between a home or hospital birth. The declining rate of midwife-led home births alongside the recent debate on safety of home births in the Netherlands, however, suggest an association of choice of birth place with psychological factors related to safety and risk perception. In this study associations of pregnancy related anxiety and general anxious or depressed mood with (changes in) planned place of birth were explored in low risk women in midwife-led care until the start of labour.

**Methods:**

Data (*n* = 2854 low risk women in midwife-led care at the onset of labour) were selected from the prospective multicenter DELIVER study. Women completed the Pregnancy Related Anxiety Questionnaire-Revised (PRAQ-R) to assess pregnancy related anxiety and the EuroQol-6D (EQ-6D) for an anxious and/or depressed mood.

**Results:**

A high PRAQ-R score was associated with planned hospital birth in nulliparous (aOR 1.92; 95% CI 1.32–2.81) and parous women (aOR 2.08; 95% CI 1.55–2.80). An anxious or depressed mood was associated with planned hospital birth (aOR 1.58; 95% CI 1.20–2.08) and with being undecided (aOR 1.99; 95% CI 1.23–2.99) in parous women only. The majority of women did not change their planned place of birth. Changing from an initially planned home birth to a hospital birth later in pregnancy was, however, associated with becoming anxious or depressed after 35 weeks gestation in nulliparous women (aOR 4.17; 95% CI 1.35–12.89) and with pregnancy related anxiety at 20 weeks gestation in parous women (aOR 3.91; 95% CI 1.32–11.61).

**Conclusion:**

Low risk women who planned hospital birth (or who were undecided) more often reported pregnancy related anxiety or an anxious or depressed mood. Women who changed from home to hospital birth during pregnancy more often reported pregnancy related anxiety or an anxious or depressed mood in late pregnancy. Anxiety should be adequately addressed in the process of informed decision-making regarding planned place of birth in low risk women.

## Background

Current national and international guidelines in Western countries, such as the UK and Canada, emphasize the importance for women with a low risk for pregnancy and childbirth complications to have a choice in maternity care services and in planning birth place. This is in line with the international focus to improve rates of physiologic labour and birth [1–3]. In the UK, for example, low risk women may choose to give birth in a free-standing midwifery unit or at home with midwife attendance (low-technology birth) or in an obstetric-unit (high-technology birth). Correspondingly, in the Netherlands low risk women routinely receive midwife-led care and may choose between home or short-stay hospital birth – both attended by their own midwife. If complications arise, women are transferred to an obstetrician-led unit.

Despite the availability of low-technology birth services to low risk women, in most Western countries births usually take place in hospital nowadays. For example, in the UK only 8% of women gave birth at home or in a midwifery unit in 2007 [4]. The highest rates of home birth can still be found in the Netherlands, although the Dutch home birth rate has significantly declined as well from 32% between 2001 and 2003 to 21% between 2010 and 2012 [5]. This decline may be associated with the growing demand of medical pain relief which is only available in obstetrician-led care [6, 7] and with a change in policies regarding maternal risk factors such as high BMI leading to more women identified as having a medium- or high risk profile [8]. Another important factor, however, might be the continuing debate on the safety of home birth in the Netherlands [9, 10]. Although evidence regarding the association of perinatal mortality or morbidity rates with planned place of birth has long been rather conflicting and hampered by methodological shortcomings [11–14], recent large scale studies show greater consistency [15–18]. One such study in the UK found similar perinatal morbidity and mortality rates for parous women in all birth settings and for nulliparous women in obstetric and midwifery units, but poorer outcomes for nulliparous women with planned home birth [15]. In the Netherlands, no increased rates of mortality or morbidity among parous and nulliparous low risk women were found among planned home births compared to planned midwife-led hospital births [16–18]. There is evidence to suggest that low risk women who plan a home birth have a lower risk of obstetric interventions, and a higher chance to give birth spontaneously [19, 20]. Increased risk of interventions in a hospital birth setting is reflected in costs as well. A large prospective cohort study from the UK showed that planned home birth was the most cost-effective option for low risk women compared to obstetric, freestanding or alongside midwifery units, particularly for parous women [21]. In the Netherlands, an increase of costs of giving birth in hospital is (partly) covered by charging low risk women with a payment of approximately 300 euro’s for the short-stay in hospital. This information is important for women in terms of informed decision-making regarding place of birth and other intrapartum care choices such as the possibility of pain-relief [22], as well as in terms of their psychological wellbeing such as an increased risk of potentially distressing obstetric interventions depending on their planned place of birth [23].

Previous reports have also shown that a variety of other factors is associated with planned place of birth in low risk women. Socio-demographic factors such as nulliparity, younger age, ethnic minority, and lower socio-economic background characterize low risk women who more likely plan a hospital birth compared to a home birth [24–26]. Concerning attitudes of low risk women, it has been found that women who plan a hospital birth have more positive attitudes towards obstetric technology and the availability of medical pain-relief during birth [27, 28]. Women who plan a home birth, however, tend to have a more fundamental trust in the independent ability to give birth, the birth process itself, and value the privacy of the home environment and the ability to make decisions during the birth process [29–31]. Also, positive attitudes of midwives regarding home birth and a critical attitude to hospital birth for non-medical reasons have been found to explain some of the variance found in home birth rates across Dutch midwifery practices [32]. Positive attitudes towards home births among UK maternal caregivers in general, are associated with the amount of experience and confidence in managing obstetric risks in home births and with maternal caregivers’ beliefs about safety of home births [33]. Other interesting outcomes related to preferred place of birth and risk perception have been found as well. A study in the UK showed that nulliparous low risk women who preferred a low-technology birth place in early pregnancy, also received fewer ultrasounds during pregnancy compared to matched low risk controls who preferred a high-technology labour in obstetric led-care [34].

Indeed, several studies have recently shown that motivational factors relating to risk-perception and safety seem critical in the choice of low risk women to plan hospital instead of home birth [35–37]. Recently, perceptions of safety and risk were found to be the strongest determinants for choosing hospital birth in a cohort of low risk Canadian women [37]. Psychological factors, especially pregnancy related anxiety, have been found to be important predictors of pregnancy risk perception [38, 39]. The latter is of interest, since about 80% of low risk women has some worries about childbirth and about 20% experiences more intense pregnancy related fears or anxiety [40, 41]. Worries, anxiety and fear in relation to pregnancy and childbirth are, however, different concepts. Recent research shows that worries about childbirth are often described as unspecific feelings and thoughts while fear of childbirth concerns a strong feeling connected to something specific [42]. Also, more general anxiety such as trait anxiety, has been found to predict fear of childbirth [43] and should be differentiated from pregnancy related anxiety [44]. Furthermore, antenatal depression and anxiety, as well as fear of childbirth, have been found to be associated with adverse perinatal outcomes such as pre-term birth [45–47].

It is thus highly relevant that maternity care providers have information on the possible association between these psychological symptoms of low risk women and their choice in place of birth. If anxious or depressed women are more likely to choose hospital birth, health professionals can use this information to better support women or to alleviate anxiety, for example by giving evidence based information on place of birth. This may further enable women to make a well informed decision on place of birth. Very little research, however, is available on associations between psychological factors and planned place of birth. The only study we know of showed that symptoms of depression and health worries were related to a preference of low risk nulliparous women for hospital birth in obstetrician-led care [31]. As far as we are aware, no information is available on the association of psychological characteristics and the choice for midwife-led planned place of birth.

Based on the above, our first research aim was to examine the association between pregnancy related anxiety and general anxious or depressed mood with planned place of birth. We hypothesized that low risk women who plan a hospital birth will have higher levels of pregnancy related anxiety such as fear of childbirth, than women who plan a home birth. Pregnancy related anxiety and general anxiety are not the same [44] and pregnancy related anxiety also tends to influence general anxiety over the course of pregnancy [47]. We therefore expected general anxiety, to be related to planned place of birth as well. Since fear of childbirth shows temporal changes throughout pregnancy [48] and a certain proportion of low risk women changes their planned place of birth throughout pregnancy (i.e. 28% of nulliparous women in the UK [34]), our second research aim was to examine whether changes in planned place of birth throughout the course of pregnancy were associated with anxiety. We hypothesized that a change in planned place of birth from early to late pregnancy was associated with pregnancy related anxiety and an anxious or depressed mood early in pregnancy and/or with becoming anxious or depressed in late pregnancy.

## Methods

### Study design and study population

The data for the current paper were selected from the DELIVER-dataset [49]. This dynamic cohort study consisted of women who completed up to three questionnaires between their first appointment in the midwifery practice and six weeks postpartum (between September 2009 and March 2011). With purposive sampling taking into account level of urbanization, region and practice type, 34 out of the 519 midwife-led care practices in the Netherlands were approached to achieve a sample of 20 practices that were willing to participate. From these practices, a total of 7685 women participated by returning at least one questionnaire. All participating women gave informed consent. Further details on design, recruitment and non-response have been published elsewhere [49].

**Fig. 1:**
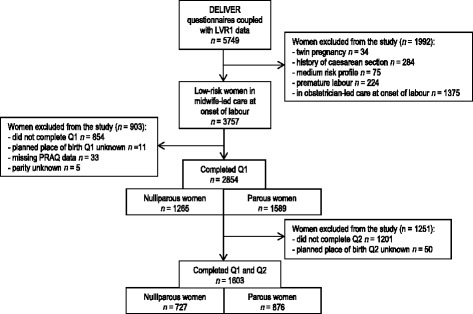
Flowchart of study sample. Q1 = questionnaire 1; Q2 = questionnaire 2

Data from the participating women in the DELIVER-study were coupled with midwife-led care data obtained from the Netherlands Perinatal Registry, the ‘Landelijke Verloskundige Registratie’ (LVR1) data [50]. Linkage succeeded in 74.8% (*n* = 5749) of all participating women (see Fig. 1). In the Netherlands, low risk women in midwife-led care are referred by their midwives to obstetrician-led secondary care, when risk factors arise during pregnancy, labour or the postpartum period based on the List of Obstetric Indications (LOI) [51]. For this study, participants who gave birth prematurely (<37 weeks) (*n* = 224) and those with a high or medium risk indication according to the LOI (for example twin pregnancy) (*n* = 393) were excluded because in the Netherlands they are not offered the choice of home birth. Information about onset of labour, premature labour and risk factors was obtained from LVR1. Furthermore, women who were referred to obstetrician-led care during pregnancy and were in obstetrician-led care at the onset of labour were also excluded (*n* = 1375). This resulted in a sample of low risk nulliparous and parous women with a singleton pregnancy, who gave birth from 37 weeks onwards who did not have an indication for obstetrician-led care and who started labour under the supervision of a primary-care midwife (*n* = 3757). For our first research aim, data from the first questionnaire administered between the first antenatal visit and before 35 weeks of gestation were used. This questionnaire contained information about planned place of birth and psychological factors relevant to this study. Women were therefore also excluded from our study when the first questionnaire was not completed and/or no data was available on planned place of birth, PRAQ-R or parity (*n* = 903). This resulted in a sample of 2,854 participating women. For our second research aim, we selected participants who, in addition to the first questionnaire, completed the second questionnaire after 35 weeks of gestation as well (*n* = 1603).

### Planned place of birth

Our primary outcome measure consisted of the question to the client where she planned to give birth. A response on this item was requested in both questionnaire one (before 35 weeks of gestation) and two (after 35 weeks of gestation). Women were able to choose from six response options; 1) ‘at home’, 2) ‘in hospital, midwife-led care’, 3) ‘in hospital, obstetrician-led care’, 4) ‘a birth centre’, 5) ‘somewhere else, specifically…’ or 6) ‘do not know yet’. Eighty-six women chose birth in hospital under obstetrician-led care (option 3) although they were in midwife-led care at the onset of labour. We included these women in the planned hospital group (midwife-led care; option 2). Probably these women planned to have medicinal pain relief as soon as labour started, for which they would have to be referred to obstetrician led care, or some might have misunderstand the question. Also, a few women planned to give birth in ‘a birth centre’ (option 4; *n* = 12). All birth centers at the time of the study were located alongside an obstetric unit in hospital. Hence, we aggregated options 3 and 4 together with option 2, i.e. planned hospital birth (midwife- led). Responses from women planning to give birth somewhere else (response option 5) were carefully screened and recoded when applicable to one of the response options. Option 6 was operationalized as ‘undecided’.

### Pregnancy related anxiety and anxious or depressed mood

The PRAQ-R, a revised and validated version of the original 58 item PRAQ scale [52], contains 10 items representing three correlated factors: fear of giving birth, fear of bearing a physically or mentally handicapped child, and concern about one's own appearance. Item responses were on a 4-point scale, ranging from “definitely true” to “definitely not true”. Good internal reliability of the subscales as well as the total score was found [44]. For the parous subsample one item from subscale ‘fear of giving birth’ had to be excluded; ‘I fear giving birth, because I have never experienced this before’ [53]. As a result, scoring ranged on the subscale ‘fear of childbirth’ and total score deviated between parous and nulliparous women. Continuous scores were calculated for all subscales and the total score. The items were summed up, resulting in possible total scores ranging from 10 to 40 for nulliparous women, and 9 to 36 for parous women. Higher scores reflect higher levels of pregnancy related anxiety. A dichotomous cut-off score of 26 and higher and 21 and higher was chosen for nulliparous and parous subsamples respectively, hereby identifying the 15% highest scoring women on the PRAQ-R total score, similar to the procedure in a recent validation study of pregnancy related anxiety measures including the PRAQ-R [54]. The PRAQ-R was assessed in the first DELIVER questionnaire only.

The EQ-6D measures health-related quality of life in 6 domains: mobility, self-care, usual activities, pain/discomfort, anxiety/depression, and cognitive functioning [55]. All dimensions are single items. For the current paper we selected the item covering the anxiety/depression domain, i.e. *How is your mood at this moment?* Response options were ‘not at all, moderately or severely anxious or depressed’, respectively. The responses were dichotomized into ‘not anxious or depressed’ or ‘anxious or depressed’. Responses on the EQ-6D were collected in both DELIVER questionnaires (Q1: before 35 weeks and Q2: after 35 weeks).

### Potential confounding factors

Background characteristics were obtained from the DELIVER-study. Maternal age was constructed using age in years at the time of completion of the DELIVER questionnaire and was subsequently divided into three categories. Gestational age at time of completion of the questionnaire was continuous (in weeks). Ethnicity was constructed using the responses on nationality in the DELIVER data. Ethnicity was dichotomously operationalized as ‘Dutch’ (when both parents are born in the Netherlands) or ‘non-Dutch’ (when at least one of the parents is born in another country). For socio-economic status status-score was calculated by the National Institute for Social Research. It is based on the mean income, employment rate, and educational level of the neighborhood determined by the woman’s postal code. The status-score was linked to the DELIVER dataset, through postal codes and it was divided in tertiles with 1 representing high socio-economic status. Pre-pregnancy BMI was calculated based on women’s reported weight before pregnancy and their height. In case of missing values, data on pre-pregnancy weight and height from the electronic records completed by midwives were used if available.

Maternal age, ethnicity and socioeconomic status were entered as possible confounders based on previous research showing a relation of these factors with both planned place of birth [16] and the risk of maternal antenatal depression and anxiety [56–59]. Maternal body mass index (BMI) was also entered as a possible confounder since a positive correlation was demonstrated between pre-pregnancy BMI and anxiety and depressive symptoms [60] and between BMI and (planned) place of birth [31].

Potential confounding influence of the obstetric factors ‘previous pregnancy loss (due to miscarriage or termination of pregnancy)’ in nulliparous women and ‘history of assisted vaginal delivery (AVD)’ and ‘previous place of birth’ in parous women was explored as part of the sensitivity analyses performed. Research for example shows that women with a certain obstetric history such as miscarriage have greater pregnancy-specific anxieties [61] and more often prefer a hospital birth in obstetric-led care [31] than pregnant women without such history. In the sensitivity analyses, we examined whether the association between anxiety and planned place of birth changed when these factors were entered in the regression model.

### Data-analysis

For descriptive purposes, percentages of background characteristics were calculated according to planned place of birth (either home, hospital or undecided) stratified by parity. Univariate differences were analysed using χ^2^ tests. We stratified our analyses by parity because nulliparous and parous are inherently different according to previous labour experiences and differ in level and content of fear (of childbirth) and anxiety [43] and planned place of birth [24]. Furthermore, background characteristics of low risk participants who were excluded from the analyses because of incomplete data (i.e. questionnaire one, *n* = 903 and two, *n* = 1251), were compared to the participants included, respectively *n* = 2854 and *n* = 1603 (see Fig. 1).

For the first research aim, a multinomial logistic regression model was conducted to estimate the association of pregnancy related- and general anxiety measures (separate independent variables) with planned place of birth as dependent variable. Planned home birth was the reference category for these comparisons. In a multivariable logistic regression model the associations were adjusted for potentially confounding factors maternal age, SES, BMI and ethnicity.

For the second research aim, binomial logistic regression analyses were performed on subsamples of nulliparous and parous women who completed the first and the second DELIVER questionnaire in order to assess associations between a change in planned place of birth from before 35 weeks to after 35 weeks gestation and a) high pregnancy-specific anxiety (a score above the PRAQ-R cut-off score); b) an anxious or depressed mood (EQ-6D) before 35 weeks gestation; and c) a change from a non-anxious or non-depressed mood (EQ-6D) before 35 weeks to anxious or depressed mood after 35 weeks of gestation. Several (‘change’) outcome variables were constructed: 1) from planned home birth to planned hospital birth (or undecided) compared with planned home birth in both early and late pregnancy and 2) from planned hospital birth to planned home birth (or undecided) compared with planned hospital birth in both early and late pregnancy. Compared to women who did not change their planned place of birth throughout pregnancy, we expected women who changed from hospital to home to be less anxious and women who changed from home to hospital birth to be more anxious, respectively. Associations for the second research aim were adjusted for SES only (three categories operationalized into two dummy-variables), due to the smaller N and the rule of thumb of a minimum of ten events in the outcome variable per covariate. We chose SES as a necessary potential confounder because of the fact that it is correlated with education, and ethnicity and because low SES is associated with higher psychological distress levels [62]. Furthermore, as shown in Table 1, both nulliparous and parous women were significantly different in SES according to planned place of birth.

**Table 1:** Baseline characteristics of low-risk women in midwife-led care according to planned place of birth

	Nulliparous *n* = 1265							Parous *n* = 1589						
	Home		Hospital		Undecided			Home		Hospital		Undecided		
Variable	*n* = 484 (38.3%)		*n* = 620 (49%)		*n* = 161 (12.7%)		p	*n* = 848 (53.4%)		*n* = 614 (38.6)		*n* = 127 (8%)		p
Maternal age							<.0001							0.139
< 25	93	19.3	99	16.0	26	16.1		33	3.9	31	5.0	5	3.9	
25–34	363	75.2	426	68.7	117	72.7		625	73.8	419	68.2	97	76.4	
> 35	27	5.6	95	15.3	18	11.2		189	22.3	164	26.7	25	19.7	
SES							<.01							<.01
High	108	22.5	151	24.4	45	28.1		241	28.5	166	27.1	38	29.9	
Medium	254	52.9	261	42.1	71	44.4		443	52.3	271	44.3	61	48.0	
Low	118	24.6	208	33.5	44	27.5		163	19.2	175	28.6	28	22.0	
Education							N.S.							N.S.
Low	58	12.0	83	13.4	23	14.3		127	15.0	118	19.3	20	15.7	
Medium	191	39.5	202	32.7	60	37.3		305	36.1	208	34.1	52	40.9	
High	235	48.6	333	53.9	78	48.4		414	48.9	284	46.6	55	43.3	
Ethnicity							<.0001							<.0001
Dutch	475	98.1	546	88.2	144	89.4		826	97.4	549	89.4	119	93.7	
Non-Dutch	9	1.9	73	11.8	17	10.6		22	2.6	65	10.6	8	6.3	
Body Mass Index							<.05							N.S.
< 18.50	3	1.2	23	3.8	8	5.0		25	3.0	16	2.6	5	4.0	
18.50–24.99	371	77.0	433	70.8	123	76.9		586	69.9	406	67.0	80	64.0	
25.00–29.99	87	18.0	122	19.9	22	13.8		179	21.4	136	22.4	28	22.4	
30.00–34.99	15	3.1	24	3.9	6	3.8		42	5.0	41	6.8	10	8.0	
> 35.00	3	0.6	10	1.6	1	0.6		6	0.7	7	1.2	2	1.6	
Previous pregnancy^a^							<.05							
Yes	50	10.4	97	15.6	20	12.4								
No	433	89.6	523	84.4	141	87.6								
Gestational age^b^							<.0001							<.0001
Mean (SD)	482	21.5/6.8	619	19.9/6.4	159	17.8/5.5		846	21.0/6.5	607	19.8/6.3	125	18.7/6.1	
Median (IQR)	482	21 (16–27)		19 (15–24)		17 (14–21)			20 (16–25)		19 (15–24)		18 (14–23)	
History of AVD														<.0001
Yes								68	8.0	108	17.6	17	13.4	
No								780	92.0	506	82.4	110	86.6	
Previous birth place														<.0001
Home								505	61.5	40	6.6	28	22.6	
Hospital, midwife-led								54	6.6	177	29.1	25	20.2	
Hospital, obstetrician-led								262	31.9	391	64.3	71	57.3	

Data shown: no. (%) of women

^a^Pregnancy loss before 24 weeks of gestation (due to miscarriage or termination of pregnancy)

^b^Gestational age at the moment of completing the first questionnaire

Additionally, sensitivity analyses were performed. First, we examined whether associations found between pregnancy anxiety and anxious or depressed mood and planned place of birth were influenced by the factors gestational age at the moment of completing the first questionnaire in nulliparous and parous women, previous pregnancy loss in nulliparous women and history of assisted vaginal delivery (AVD) and previous birth place in parous women. Second, in a separate analysis robust variance estimation was used to allow for clustering of women within midwifery practices. Third, for a small number of women, the LVR1 data showed some discrepancies for the onset of labour in midwife-led care. Therefore, we conducted sensitivity analyses for women without any discrepancies in the definition for onset of labour in midwife-led care.

All results are expressed as adjusted odds ratios with 95% confidence intervals and P values. Statistical significance was considered reached at P 2-sided < 0.05. For (multinomial and binary) logistic regression analysis we assumed that the independent variables were linearly related to the log odds. All analyses were performed with SPSS version 20.0 for Windows and Stata version 12.

## Results

Background characteristics of low risk women in midwife-led care that were excluded because of incomplete or missing data (*n* = 903; Fig. 1) differed not significantly (*p* > 0.05) from those included (*n* = 2854) according to SES and ethnicity. These women excluded were, however, significantly different (*p* < 0.05) in age (i.e. < 25, 11.3% vs. 10.1%; 25–34, 67.1% vs. 71.8%; > 35; 21.5% vs. 18.2%, respectively) and parity (i.e. nulliparous 51.3% vs. 44.3%, respectively). Nulliparous women (*n* = 2,854) completed the first DELIVER questionnaire at a mean of 20.2 (SD 6.6) weeks gestation and parous women at 20.4 (SD 6.4) weeks gestation. Of the nulliparous women, 484 (38.3%) planned to give birth at home, 620 (49%) in hospital, and 161 (12.7%) were undecided. Of the parous women, 847 (53.3%) intended to give birth at home, 614 (38.7%) in hospital, and 127 (8.0%) were undecided. From the sample of 2854 low risk women, we selected women (*n* = 1603; Fig. 1) who also completed questionnaire 2 in the third trimester of pregnancy (i.e. at a mean of 37.4 weeks (SD 1.4) for both nulliparous and parous women). Background characteristics (age, socioeconomic status, parity and ethnicity) of low risk women in midwife-led care that were excluded because of incomplete or missing data on questionnaire 2 (*n* = 1,251; Fig. 1) differed significantly (*p* < .05) from those included in the analyses (*n* = 1603) in ethnicity (i.e. 9.8% vs. 4.5% of non-Dutch origin, respectively), SES (i.e. low 23.1% vs. 28.8%; medium 47% vs. 48.5%; high 29.9% vs. 22.7%, respectively), and age (i.e. < 25, 13.9% vs. 7%; 25–34, 69.1% vs. 73.9%; > 35; 17% vs. 19.1%, respectively).

### Background characteristics and planned place of birth

Table 1 shows that nulliparous and parous women who reported in the first questionnaire that they were planning to give birth at home, were more often of Dutch origin, had a medium socio-economic status and had a higher gestational age compared to women in the other two groups (i.e. planning a hospital birth or being undecided regarding planned birth place). Nulliparous women planning to give birth at home were more often younger and less often had a previous pregnancy loss than women in the other two groups. Parous women planning to give birth at home less often had a history of AVD compared to women in the other two groups. In general, nulliparous and parous women undecided about planned place of birth were somewhere in-between the women who planned a home birth and women who intended to give birth at a hospital according to background characteristics. The majority of parous women in the planned home birth group previously gave birth at home as well. Of the parous women currently planning a hospital birth, approximately one-third previously gave birth in hospital in midwife-led care as well, while two-third previously gave birth in obstetrician-led care. The majority of parous women undecided about place of birth previously gave birth in hospital in obstetrician-led care.

### Pregnancy related anxiety and general anxious or depressed mood with planned place of birth

Table 2 shows that parous women who were anxious or depressed (EQ-6D) had significantly increased odds for planning a hospital birth and for being undecided relative to planning a home birth. Both nulliparous and parous women with a pregnancy related anxiety level (PRAQ-R total symptom level) within the 15% highest scores had significantly increased odds for planning a hospital birth relative to planning a home birth. Furthermore, with every unit increase on the PRAQ total symptom scale, the odds were significantly elevated for planning a hospital birth or for being undecided, i.e. 6 to 8%, respectively, relative to planned home birth. Similarly, on the PRAQ subscales ‘fear of childbirth’ and ‘fear of a handicapped child’, nulliparous and parous women had significantly elevated adjusted odds for planning a hospital birth and for being undecided, all relative to planning a home birth. For subscale ‘fear of own appearance’, the adjusted odds were only significantly elevated for nulliparous and parous women who intended to give birth in hospital.

**Table 2:** Association between planned place of birth and pregnancy anxiety and general anxious/depressed mood

	Nulliparous *n* = 1265			Parous *n* = 1589		
	Planned home birth (*n* = 484/38.3%)	Planned hospital birth (*n* = 620/49%)	Undecided (*n* = 161/12.7%)	Planned home birth (*n* = 847/53.3%)	Planned hospital birth (*n* = 614/38.6%)	Undecided (*n* = 127/8%)
EQ-6D anxious/depressed						
No. (%)	69 (14.3)	123 (19.8)	32 (19.9)	130 (15.3)	143 (23.3)	34 (26.8)
aOR (95% CI)	Reference	1.38 (0.98–1.94)	1.43 (0.89–2.30)	Reference	1.58 (1.20–2.08)^c^	1.99 (1.23–2.99)^c^
PRAQ total > cut off^a^						
No. (%)	47 (9.7)	110 (17.7)	25 (15.5)	94 (11.1)	139 (22.6)	24 (18.9)
aOR (95% CI)	Reference	1.92 (1.32–2.81)^c^	1.51 (0.88–2.58)	Reference	2.08 (1.55–2.80)^d^	1.62 (0.97–2.71)
PRAQ total score						
Mean (SD)	20.3 (4.3)	21.5 (4.8)	21.7 (4.4)	16.1 (3.8)	17.5 (4.3)	17.5 (4.3)
aOR (95% CI)	Reference	1.06 (1.03–1.09)^d^	1.07 (1.03–1.11)^c^	Reference	1.08 (1.05–1.11)^d^	1.08 (1.03–1.13)^c^
PRAQ fear childbirth						
Mean (SD)	6.5 (1.8)	7.1 (2.1)	7.2 (2.1)	3.4 (1.1)	3.9 (1.3)	3.8 (1.3)
aOR (95% CI)	1.0	1.17 (1.09–1.24)^d^	1.20 (1.09–1.32)^d^	Reference	1.34 (1.23–1.47)^d^	1.30 (1.12–1.52)^c^
PRAQ fear handicapped child						
Mean (SD)	7.8 (2.2)	8.1 (2.3)	8.4 (2.2)	7.4 (2.1)	7.9 (2.4)	8.0 (2.4)
aOR (95% CI)	Reference	1.06 (1.01–1.13)^b^	1.11 (1.02–1.20)^b^	Reference	1.09 (1.04–1.14)^c^	1.10 (1.01–1.20)^b^
PRAQ fear own appearance						
Mean (SD)	6.0 (1.9)	6.2 (2.0)	6.1 (1.9)	5.4 (1.9)	5.8 (2.1)	5.7 (1.9)
aOR (95% CI)	Reference	1.07 (1.01–1.14)^b^	1.03 (0.94–1.14)	Reference	1.09 (1.03–1.15)^c^	1.09 (0.99–1.20)

aOR: odds ratios were adjusted for age, SES, BMI and for ethnicity

Odds ratios are calculated relative to planned home births (*n* = 1265, nulliparous women; *n* = 1589 parous women)

^a^nulliparous women PRAQ total score of 26 and higher; parous women PRAQ total score 21 and higher

^b^ = <.05 ^c^ = <.01 ^d^ = < .0001

### Change from early to late pregnancy in planned place of birth

Of the women who completed both questionnaires (i.e. before 35 weeks and after 35 weeks gestation; *N* = 727 nulliparous and *N* = 876 parous), 81.6% nulliparous women and 89.2% parous women (Fig. 1), did not change their initially planned place of birth later in pregnancy. Table 3 shows that nulliparous women who changed their initial plan of home birth to hospital birth after 35 weeks, significantly more often report an anxious or depressed mood after 35 weeks while not being anxious or depressed at 20 weeks, relative to nulliparous women who did not change their planned home birth (OR 4.17, CI 1.35–12.89). Parous women who changed their initial plan of home birth into a hospital birth after 35 weeks, more often had a high level of pregnancy related anxiety at 20 weeks than women who did not change their planned place of birth at home (OR 3.91, CI 1.32–11.61). Among nulliparous and parous women no significant associations were found between anxiety scores based on PRAQ-R and EQ-6D and changing from planned hospital to planned home birth (Tables 3 and 4, respectively).

**Table 3:** Associations between changes in planned place of birth and pregnancy anxiety and general anxious/depressed mood

Nulliparous women				
	Nulliparous (*n* = 633)^a^			
	Home – home (*n* = 279/38.4%)	Home – hospital^b^ (*n* = 18/2.5%)	Hospital-hospital (*n* = 300/41.3%)	Hospital – home^c^ (*n* = 36/5%)
PRAQ total > cut off or anxious/depressed 20wks				
No (%)	21 (7.5)	1 (5.6)	47 (15.7)	6 (16.7)
aOR (95% CI)	Reference	0.67 (0.08–5.32)	Reference	1.09 (0.43–2.79)
Anxious/depressed at 20 wks. (EQ6D)				
No (%)	30 (10.8)	3 (16.7)	49 (16.3)	7 (19.4)
aOR (95% CI)	Reference	1.61 (0.44–5.97)	Reference	1.17 (0.48–2.85)
Change not anxious/depressed at 20 wks. to anxious/depressed at 37 wks. (EQ6D)				
No (%)	24 (8.7)	5 (27.8)	29 (9.7)	4 (11.1)
aOR (95% CI)	Reference	4.17 (1.35–12.89)^d^	Reference	1.04 (0.34–3.21)

ORs adjusted only for two dummy-categories SES due to the smaller N and the rule of thumb of a minimum of ten events in the outcome variable per covariate

^a^Excluded: *n* = 94 clients undecided regarding planned place of birth at 20 wks

^b^Included: *n* = 5 clients who changed from home to undecided

^c^Included: *n* = 5 clients who changed from hospital to undecided

^d^ = <.05

**Table 4:** Associations between changes in planned place of birth and pregnancy anxiety and general anxious/depressed mood

Parous women				
	Parous (*n* = 814)^a^			
	Home-home (*n* = 492/56.2%)	Home – hospital^b^ (*n* = 17/1.9%)	Hospital-hospital (*n* = 282/32.2%)	Hospital – home^c^ (*n* = 23/2.6%)
PRAQ total > cut off or anxious/depressed 20wks				
No (%)	48 (9.8)	5 (29.4)	57 (20.2)	5 (21.7)
aOR (95% CI)	Reference	3.91 (1.32–11.61)^d^	Reference	0.24 (0.03–1.83)
Anxious/depressed at 20 wks. (EQ6D)				
No (%)	67 (13.6)	4 (23.5)	57 (20.2)	5 (21.7)
aOR (95% CI)	Reference	1.96 (0.62–6.21)	Reference	0.98 (0.10–2.82)
Change not anxious/depressed at 20 wks. to anxious/depressed at 37 wks. (EQ6D)				
No (%)	57 (11.6)	3 (17.6)	38 (13.5)	1 (4.3)
aOR (95% CI)	Reference	1.65 (0.46–5.94)	Reference	1.08 (0.38–3.08)

ORs adjusted only for two dummy-categories SES due to the smaller N and the rule of thumb of a minimum of ten events in the outcome variable per covariate

^a^Excluded: n = 62 clients undecided regarding planned place of birth at 20 wks

^b^Included: n = 3 clients who changed from home to undecided

^c^Included: n = 13 clients who changed from hospital to undecided

^d^ = <.05

### Sensitivity analyses

Additional analyses showed that gestational age at the moment of completing the first questionnaire in nulliparous and parous women, loss of previous pregnancy due to miscarriage or termination of pregnancy in nulliparous women and obstetric history of AVD and previous place of birth in parous women, did not moderate the presented associations between pregnancy related- and general anxiety and depressed mood with planned place of birth (Table 2). More specifically, associations remained unchanged when adding these factors to the multinomial regression models. Furthermore, multinomial logistic regression analyses were performed only in the subsample of women without any discrepancies regarding onset of labour (*n* = 116 women with discrepancies were left out). Results for planned place of birth and pregnancy related anxiety and general anxious or depressed mood also remained unchanged (data not shown). Taking account of clustering of women within each midwifery practice, also yielded similar results (data not shown).

## Discussion

The most salient finding of our study is that women planning a hospital birth and those who were undecided about their planned place of birth, reported higher levels of pregnancy related anxiety at 20 weeks gestation than women who planned a home birth. Among parous women, an anxious or depressed mood was only significantly more often reported by women planning a hospital birth and those who were undecided about their planned place of birth compared to women who planned a home birth. Overall, the vast majority of women did not change their initially planned place of birth later in pregnancy. A change from planned home birth around 20 weeks to hospital birth after 35 weeks pregnancy was, however, associated with a change from a non-anxious or depressed to an anxious or depressed mood later in pregnancy in nulliparous women and with a high level of pregnancy related anxiety around 20 weeks pregnancy in multiparous women.

An important strength of our study is the large sample size that gave us the ability to analyze several subgroups and to adjust for potential confounders. The only previous study into psychological symptomatology and planned place of birth showed unadjusted analyses [31]. We cannot rule out some residual confounding completely, but since the potential confounding factors that were included only had little impact on the associations, these residual factors are unlikely to change the associations to a great extent. Furthermore, the two prospective measurements of planned place of birth during pregnancy made it possible to assess whether women tend to change their mind on planned place of birth and whether such a change is related to an anxious or depressed mood. Selecting women who started labour in midwife-led care and excluding women with medical risk factors to rule out potential selection bias, is a clear strength as well. Although these data could only be obtained through linkage with the Dutch Perinatal Registry which might be a limitation, sensitivity analyses excluding women with discrepancies yielded similar results. A limitation regarding our study sample is that women who had to be excluded from our original data-set because of missing data were more often nulliparous and women who were lost after the first questionnaire assessment had more often a lower SES, non-Dutch ethnic background and younger age. However, it seems highly unlikely that the association between anxiety and mood and planned place of birth would be in the opposite direction in these non-responders. Also, there is evidence from a large epidemiological longitudinal study that this type of attrition does not necessarily influence the results [63]. Nevertheless, we cannot rule out that this type of attrition has some effect on generalizability of our findings. Limitations regarding measurement-instruments are that the EQ-6D is a generic descriptive measure based on a self-reported three-level multiple choice question, which is minimal and not detailed enough to examine specific aspects of anxiety and depression. Another limitation of the EQ6D is that this anxiety/depression dimension has not been validated in a pregnant population [55]. Moreover, although the PRAQ-R has been shown to have good content and construct validity of pregnancy specific anxiety in nulliparous and parous women [47, 53], a measure specifically assessing fear of childbirth such as the Wijma-Delivery Expectancy Scale (W-DEQ) might have given a deeper understanding of which aspects of fear of childbirth particularly relate to planned place of birth [43, 64].

Interestingly, our findings only partly agree with those of Van Haaren-ten Haken et al. [31] who found planned hospital birth to be associated with higher levels of depressive symptomatology and health-worries only in low risk women receiving obstetrician-led care during pregnancy, while pregnant women in midwife-led care did not show these differences in symptomatology for planned place of birth either at home or in hospital. However, they found a similar trend of higher symptom levels in women in midwife-led care planning a hospital birth compared to home birth [31]. Therefore, this discrepancy in findings might well be due to our larger sample size. Our findings correspond with recent research showing that women in midwife-led care who decide to plan a hospital birth or who have difficulty in choosing their place of birth, determine their choice largely on their perceptions of risk and safety [37]. Risk perception during pregnancy, a factor shown to affect the choice regarding place of birth [34–39], might explain the associations found in our study between pregnancy related anxiety and general anxious or depressed mood with planned place of birth in hospital. In fact, recent research has indicated that compared to other significant predictors such as maternal or gestational age, pregnancy related anxiety was the strongest predictor of risk perception during pregnancy, at least in nulliparous women [38].

In the current study both nulliparous and parous women with higher levels of pregnancy specific anxiety and fear of childbirth more often planned a hospital birth or were undecided. The source of pregnancy related fears, however, might differ between nulliparous and parous women. High trait anxiety, for instance, has been found to be an important predictor of fear of childbirth in nulliparous women [43], while in parous women a previous negative birth experience has been shown to be a strong predictor [65, 66]. Interestingly, the majority of parous women in our study previously gave birth in obstetrician-led care and thus had a greater likelihood of having experienced obstetric interventions and having experienced fear of childbirth [27, 65]. Associations between anxiety and planned place of birth in parous women remained unchanged, however, when a history of assisted vaginal delivery was additionally controlled for. Although fear for the child’s health was the commonest fear reported in a large cohort study of pregnant women [67], fear of bearing a handicapped child was less strongly associated with planned hospital birth that the fear of giving birth. This might be another indication that the association found between pregnancy anxiety, e.g. fear of giving birth, and planned place of birth, is at least partly a reflection of issues that concern perception of risk and safety.

Our findings are in line with other recent findings showing that it is not unlikely for low risk women, in particular nulliparous women, to change their planned place of birth throughout pregnancy [34]. Our results show that a negative change in mood (i.e. anxious/depressed) from earlier to late pregnancy is related to a change from a planned home- to hospital birth in late pregnancy in nulliparous women. Interestingly, parous but not nulliparous women with a high total PRAQ-R score at 20 weeks gestation more often changed their plan from home- to hospital birth. This adds new insights to previous research demonstrating that anxiety levels may fluctuate within individuals from early to late pregnancy [48]. We did not find any significant changes in anxiety related to a change in planned hospital- to home birth.

With regard to background characteristics associated with planned place of birth, our findings generally agree with previous research [16, 31]. Contrary to previous findings [16, 68], however, nulliparous women planning a hospital birth were more often older than women in the planned home birth group. As suggested in previous research [31], this discrepancy is likely caused by the fact that other studies used samples including both nulliparous and parous women, with the latter being more often older and more often to plan a home birth [16, 68].

Our findings are of interest for maternity care providers who may be unaware of the association of (pregnancy related) psychological symptomatology and planned place of birth. Maternity care providers should explore any pregnancy related or general distress and should acknowledge its role during the process of decision-making regarding planned place of birth. They might also consider referral to antenatal psychological and childbirth educational interventions [69, 70] that seem promising for women with (a higher risk of) maternal distress [71]. This may reduce the influence of anxiety on the choice of place of birth. In addition, birth outcomes may be improved as low birth weight and fetal growth restriction are, to some extent, related to maternal anxiety [46].

## Conclusion

In conclusion, this study demonstrated that in low risk nulliparous and parous women, pregnancy related anxiety and general anxious and depressed mood (in parous women only) are significant factors in planning place of birth, adjusted for potential confounders. The majority of low risk women did not change their planned place of birth throughout pregnancy but nulliparous women who reported an anxious or depressed mood in late pregnancy and parous women with a high pregnancy related anxiety more often changed planned birth place from home to hospital. Presence of maternal distress during pregnancy, either in general or related to childbirth, should be carefully explored and adequately addressed in the process of informed decision-making regarding planned place of birth. Future studies should assess whether taking into account these psychological factors also results in more effective decision-making and whether this positively affects labour satisfaction rates and pre- and postnatal anxiety levels.

## Abbreviations

aOR: adjusted odds ratio
AVD: Assisted vaginal delivery
BMI: Body mass index
CI: Confidence interval
DELIVER: Data EersteLIjns VERloskunde
EQ-6D: EuroQol-6Dimensions
LOI: List of obstetric indications
LVR1: Landelijke Verloskundige Registratie 1
PRAQ-R: Pregnancy related anxiety questionnaire-revised
SD: Standard deviation
SES: Socioeconomic status
UK: United Kingdom
